# A 76-week real-world multidimensional analysis of guselkumab in moderate to severe plaque psoriasis: a retrospective cohort study based on Chinese clinical practice standards

**DOI:** 10.3389/fimmu.2026.1684996

**Published:** 2026-01-27

**Authors:** Runlu Hu, Qian Li, Xinli Liu, Yulian Hu, Yun Pan, Chuan Liu, Peilin Lu, Xiaoli Chen, Jianxia Xiong, Jingbo Zhang, Kun Huang

**Affiliations:** Department of Dermatology, The First Affiliated Hospital of Chongqing Medical University, Chongqing, China

**Keywords:** guselkumab, metabolic indicators, PASI 100 predictor, plaque psoriasis, real-world evidence

## Abstract

**Background:**

Guselkumab, a monoclonal antibody targeting IL-23p19, has shown durable efficacy and safety in global phase III trials and real-world studies. However, in Chinese populations, comprehensive real-world evidence regarding its effects across disease severities, body areas, and metabolic parameters remains limited.

**Objective:**

To conduct a multidimensional evaluation of guselkumab in moderate to severe plaque psoriasis based on Chinese clinical practice standards.

**Methods:**

This retrospective analysis enrolled 90 patients receiving guselkumab and assessed efficacy from multiple dimensions, along with adverse events, drug survival, and metabolic indicators. Composite indices were used to evaluate coronary artery disease (CAD) and insulin resistance risk.

**Results:**

Guselkumab showed sustained efficacy over 76 weeks in Chinese patients. Body area analysis showed significantly faster clearance of head lesions, with 40.0% of patients achieving complete clearance at week 4, compared to only 16.0% for lower extremities (*P* < 0.01). Baseline disease severity and switching to guselkumab due to inadequate response to prior biologics did not significantly affect treatment outcomes (*P* > 0.05). Predictors of PASI 100 at week 52 included normal BMI (OR = 6.10, 95% CI: 1.54–23.96), no phototherapy history (OR = 0.17, 95% CI: 0.04–0.71), and biologic-naïve status (OR = 0.14, 95% CI: 0.03–0.62). Mean fasting blood glucose (FBG) increased from 5.51 (0.74) to 5.65 (0.75) mmol/L at week 52 (*P* = 0.02). The Within Normal Limits (WNL) subgroup showed transient elevations in total cholesterol (week 28, *P* = 0.049) and triglycerides (week 28, *P* = 0.009). Although these indicators increased, they remained within the normal range. The Abnormal Without Treatment (AWT) subgroup exhibited decreased uric acid (UA) at week 52 (471.00 ± 51.06 to 418.72 ± 64.35 μmol/L; *P* = 0.004). Composite indices indicated no increased CAD or insulin resistance risk. Injection site reactions (32.2%) were the most common adverse events. Drug survival was 78.9% at 76 weeks.

**Conclusion:**

This study confirms the durable efficacy and safety of guselkumab in Chinese patients with plaque psoriasis, with early response heterogeneity favoring the head. Treatment did not increase CAD or insulin resistance risk. Normal BMI and biologic-naïve status were associated with superior efficacy, supporting precision management strategies that integrate cutaneous and systemic monitoring.

## 1 Introduction

Psoriasis is an immune-mediated chronic inflammatory disease affecting 2%–4% of the global population (1), imposing significant burdens through skin lesions, reduced quality of life, and multisystem comorbidities, including metabolic syndrome and cardiovascular disease (2–4). Its pathogenesis involves dysregulated IL-23/Th17 signaling, driving keratinocyte hyperproliferation and inflammation (5–7).

Guselkumab, the first monoclonal antibody targeting the IL-23 p19 subunit (2, 8),was approved by the FDA in 2017 and has emerged as a cornerstone therapy for moderate-to-severe plaque psoriasis. By selectively neutralizing IL-23, it disrupts downstream inflammatory cascades, offering sustained disease control and improved quality of life (9). Phase III trials and growing real-world evidence across diverse populations have validated its efficacy and safety (10–15). The VOYAGE 1 and VOYAGE 2 trials established guselkumab’s efficacy in moderate-to-severe plaque psoriasis, with long-term follow-up studies confirming sustained responses at 52 weeks (10). Real-world studies have further validated guselkumab’s effectiveness in diverse clinical settings (12, 13). However, knowledge gaps persist regarding deeper exploration of guselkumab efficacy, such as response heterogeneity across baseline disease severities, body areas, biologic-switching populations, and predictors of long-term response.

The association between psoriasis and metabolic disorders is well established (4). Patients with psoriasis exhibit higher rates of dyslipidemia, impaired glucose tolerance, and hyperuricemia than the general population (4, 16–18), attributable to chronic inflammation (19, 20). Guselkumab’s impact on metabolic parameters remains controversial, yet clinically important. The triglyceride–glucose (TyG) index and coronary artery disease (CAD) risk indices are composite markers integrating lipid and glucose profiles to assess cardiovascular risk, and compared with traditional indicators, these composite indices show better evaluative value (21, 22). Previous studies have explored the effects of biologic agents on metabolic parameters but have reported contradictory results (23). In Chinese cohorts, real-world data on metabolic parameters and TyG/CAD indices in guselkumab-treated patients remain scarce, necessitating regional validation.

This 76-week retrospective cohort study comprehensively evaluates the efficacy of guselkumab across different body areas and disease severities, as well as its safety, drug survival, and metabolic effects in Chinese patients with moderate-to-severe plaque psoriasis. By incorporating regional PASI dynamics and composite metabolic and cardiovascular risk indices, this work provides confirmatory and extended real-world evidence from a Chinese clinical setting, supporting treatment strategies that integrate cutaneous response with systemic homeostasis monitoring.

## 2 Methods

### 2.1 Study participants

This single-center retrospective cohort study enrolled patients with psoriasis treated with guselkumab at the outpatient clinic of the First Affiliated Hospital of Chongqing Medical University between January 2022 and December 2023. Exclusion criteria were: 1) age < 18 years; 2) non-plaque psoriasis subtypes, with the sole exception of plaque psoriasis coexisting with psoriatic arthritis; 3) baseline Psoriasis Area and Severity Index (PASI) score < 3; 4) use of systemic psoriasis therapies (including acitretin, methotrexate, and phototherapy) during the study period or within 3 months before enrollment; and (5) incomplete clinical data.

### 2.2 Study methods

The study enrolled 90 patients with moderate-to-severe plaque psoriasis who received guselkumab. Baseline clinical and demographic data were collected. Disease severity and treatment response were evaluated using the Psoriasis Area and Severity Index (PASI) at baseline and at 4, 12, 28, 52, and 76 weeks after treatment. The percentage of skin lesion improvement was statistically analyzed by body site: head, trunk, upper extremities, and lower extremities. For the analysis of metabolic indicators, including total cholesterol (TC), triglycerides (TG), high-density lipoprotein cholesterol (HDL-C), low-density lipoprotein cholesterol (LDL-C), fasting blood glucose (FBG), and uric acid (UA), patients diagnosed with relevant metabolic diseases and treated with medication at baseline were excluded. For patients who initiated medications known to affect the parameters of interest during the follow-up period, only metabolic data obtained before medication initiation were included, and medication use was recorded. Patients with one or more baseline metabolic indicators and corresponding follow-up data at any of the 12-, 28-, 52-, or 76-week time points were selected to compare changes in each indicator before and after guselkumab treatment. According to baseline indicator levels, patients were stratified into the Within Normal Limits (WNL) group and the Abnormal Without Treatment (AWT) group for subgroup analysis. Meanwhile, CAD risk indices, including the atherogenic index of plasma (AIP), atherogenic index (AI), and lipoprotein combine index (LCI), as well as the insulin resistance risk index including the triglyceride–glucose (TyG) index, were used to assess risk changes before and after treatment. Adverse events during treatment were recorded simultaneously. Referring to the criteria of a previous study (24), treatment interruption of up to 120 days (twice the conventional treatment interval) was allowed. Interruption exceeding this period was considered drug discontinuation, with discontinuation time and reasons recorded.

### 2.3 Statistical analysis

Descriptive statistics were performed for baseline demographic characteristics, disease features, comorbidities, and previous treatment history. Categorical variables were expressed as percentages (%), and continuous variables were described using mean ± standard deviation (SD). Intergroup comparisons of categorical variables were conducted using the chi-square test or Fisher’s exact test, while the independent samples t-test or Mann–Whitney U test was used for continuous variables. Patients who achieved PASI 100 at 52 weeks of treatment were included, and variables with *P* < 0.05 in univariate analysis were subjected to multivariate logistic regression to calculate odds ratios (OR) and 95% confidence intervals (CI). Furthermore, Cox hazard regression was conducted to further investigate associations identified in multivariable logistic regression; follow-up time was 76 weeks or until achievement of PASI 100. For comparisons of indices between baseline and 12, 28, 52, and 76 weeks after treatment, the paired t-test was used for parameters conforming to a normal distribution, and the Wilcoxon signed-rank test was used for non-normally distributed data. Drug retention rate was analyzed using the Kaplan–Meier method. All statistical analyses were performed using R (4.4.1) and SPSS (26.0). A *P* < 0.05 was considered statistically significant.

## 3 Results

### 3.1 Population characteristics

A total of 90 patients were enrolled, and their demographic and clinical characteristics are shown in **Table 1**. The mean baseline PASI score of the entire cohort was 11.3 ± 7.0, among which 57.8% (52/90) had moderate plaque psoriasis (PASI ≥3 and <10, China) (52), and 42.2% (38/90) had severe plaque psoriasis (PASI ≥10, China). Nineteen (21.1%) patients switched to guselkumab due to insufficient response to previous biologics (failure to achieve PASI 50 after 12 weeks of treatment). Previously used biologics included secukinumab (n = 8, 42.1%), ustekinumab (n = 5, 26.3%), ixekizumab (n = 3, 15.8%), adalimumab (n = 2, 10.5%), and certolizumab pegol (n = 1, 5.3%).

**Table 1:** Baseline demographic characteristics, disease characteristics, previous treatments, and comorbidities of the study population.

Characteristic	Subgroup	Statistics
Age, Mean ± SD, years		46.5 ± 14.7
Age of onset, Mean ± SD, years		31.0 ± 14.9
Gender, n (%)	Men	57(63.3%)
	Female	33(36.7%)
BMI(Kg/m^2^), n (%)		24.7 ± 5.5
	Normal(<25)	58(64.4%)
	Overweight(25 to <30)	24(26.7%)
	obese(≥30)	8(8.9%)
Disease duration, Mean ± SD, years		15.5 ± 12.3
Baseline PASI, Chinese Standard, n (%)		11.3 ± 7.0
	Moderate (≥3 and <10)	52(57.8%)
	Severe (≥10)	38(42.2%)
Cigarette use, n (%)	Never	57(63.3%)
	Current or ex-smoker	33(36.7%)
Psoriatic arthritis, n (%)	Without	88(97.7%)
	With	2(2.2%)
Family history of psoriasis, n (%)	No	87(96.6%)
	Yes	3(3.3%)
Comorbidity		26(28.9%)
Cardiovascular disease, n (%)	No	88(97.7%)
	Yes	2(2.2%)
Hypertension, n (%)	No	80(88.9%)
	Yes	10(11.1%)
Diabetes, n (%)	No	83(92.2%)
	Yes	7(7.8%)
Dyslipidemia, n (%)	No	85(94.4%)
	Yes	5(5.6%)
Liver disease, n (%)	No	88(97.7%)
	Yes	2(2.2%)
Prior treatments		
Conventional systemic agents, n (%)	No	62(68.9%)
	Yes	28(31.1%)
Phototherapy, n (%)	No	63(70.0%)
	Yes	27(30.0%)
Biologic therapy, n (%)	No	63(70.0%)
	Yes	27(30.0%)

BMI, body mass index (calculated as weight in kilograms divided by height in meters squared); PASI, psoriasis area and severity index; SD, standard deviation.

### 3.2 Clinical efficacy

**Figure 1:**
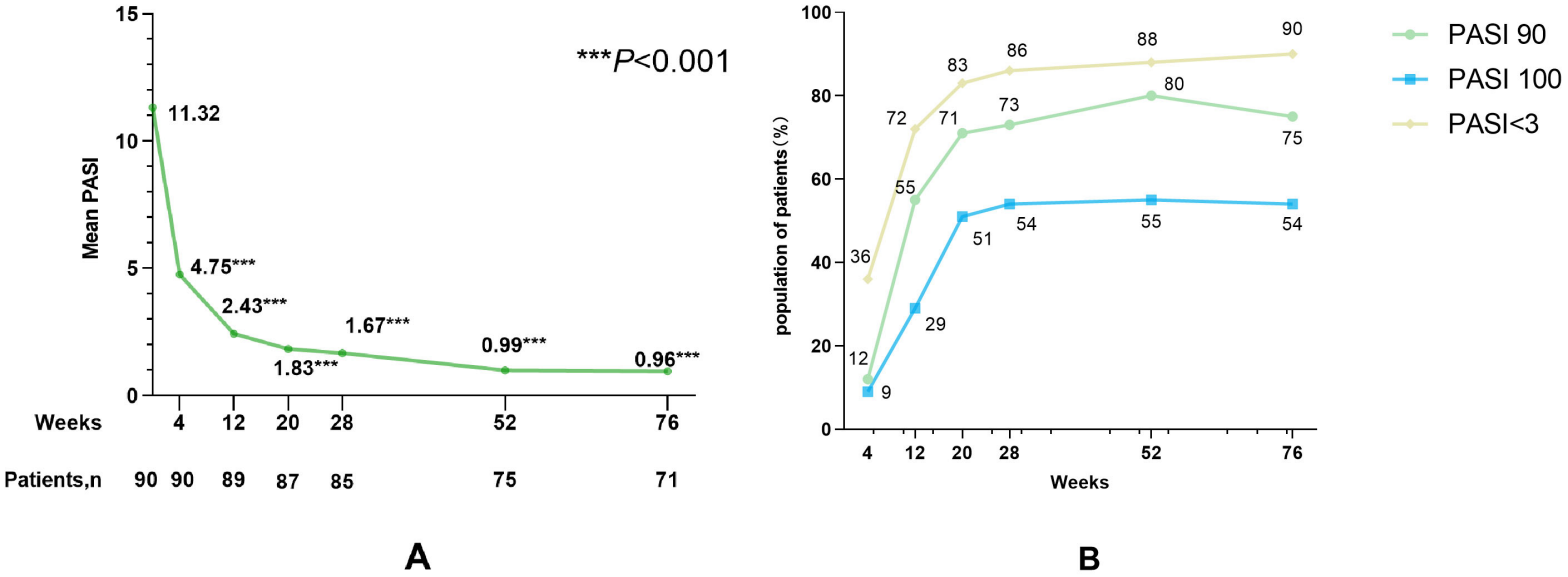
**(A)** Trend of mean PASI observed at week 0, 4, 12, 20, 28, 52, and 76. **(B)** Proportion of patients achieving PASI<3, PASI 90, and PASI 100 at week 0, 4, 12, 20, 28, 52, and 76.

**Figure 2:**
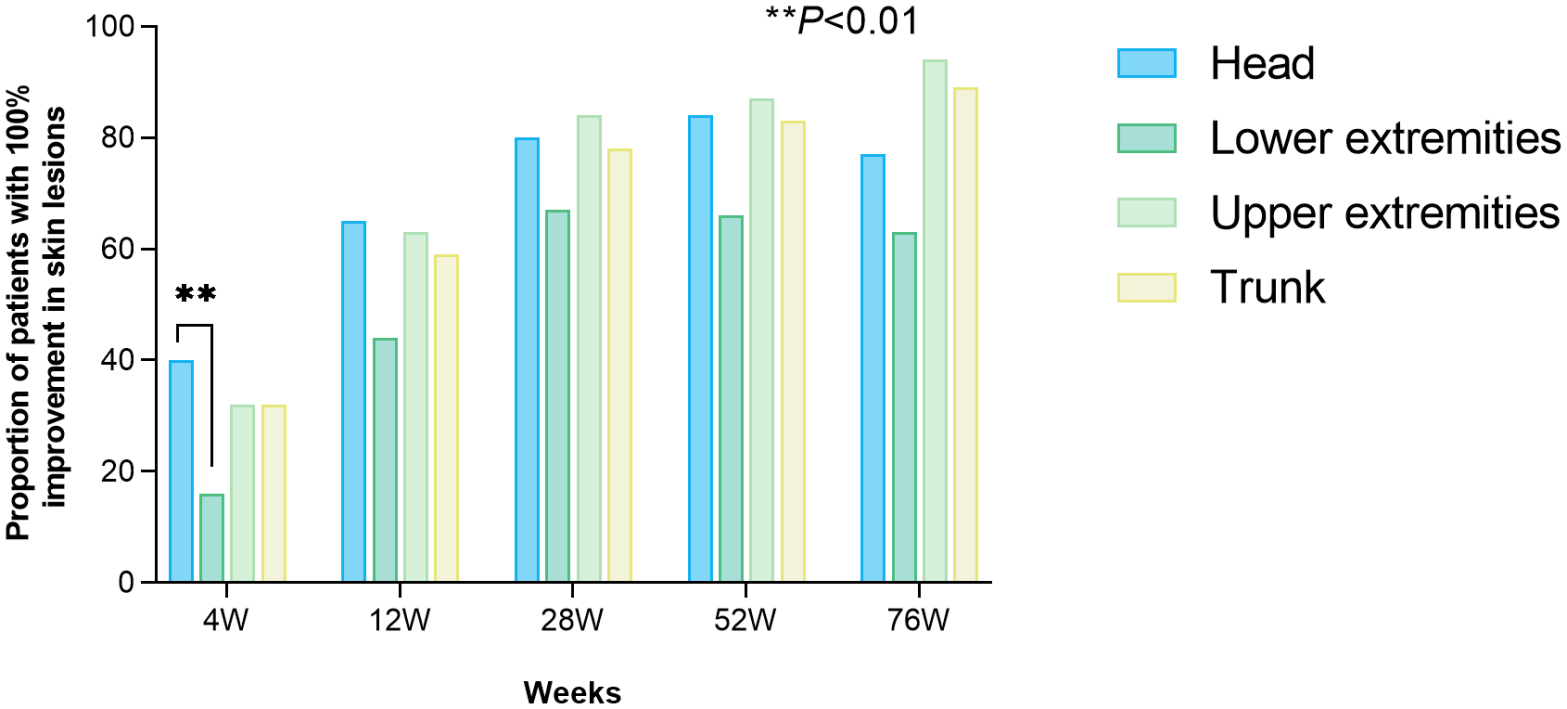
Population of patients with 100% improvement in skin lesions of head, lower extremities, upper extremities, and trunk, respectively.

Mean PASI values at all follow-up time points showed significant reductions from baseline (all *P* < 0.001; **Figure 1A**). PASI response rates at each time point are shown in **Figure 1B**. Kaplan–Meier curves for time to PASI 90 and PASI 100 are provided in **Supplementary Figure 1**. Among patients with moderate baseline disease severity (PASI ≥3 and <10), PASI 100 response rates at 4, 12, 20, 28, 52, and 76 weeks showed no significant differences compared with patients with severe baseline disease severity (PASI ≥10) (**Supplementary Figure 2**). In 19 patients who failed to achieve PASI 50 after 12 weeks of prior biologic therapy, the PASI 100 response rate following guselkumab did not differ significantly compared with 19 controls randomly selected and matched 1:1 based on age ( ± 2 years), sex, baseline PASI, disease duration, and BMI (all *P* > 0.05) (**Supplementary Figure 3**). Response analysis by body area showed that the head exhibited the fastest early response, with 40% of patients achieving complete skin lesion clearance at 4 weeks, whereas the lower extremities showed a relatively delayed response, with 16% of patients achieving clearance at that time point (*P* < 0.01; **Figure 2**). No other statistically significant differences were detected among the four body areas during follow-up. **Supplementary Figure 4** presents regional PASI trajectories using a line plot. Additionally, a linear mixed-effects model was fitted to compare progression rates of lesion improvement across body regions, with time, body region, and their interaction included as fixed effects, and patient-specific random intercepts (**Supplementary Table 1**). The model showed a significant main effect of time (*P* = 0.001), indicating increasing lesion improvement rates over time across all regions. Baseline PASI was significantly lower in the lower extremities compared with the head (*P* = 0.012); no differences were observed for the trunk or upper extremities. The time-by-region interaction was non-significant for all regions (all *P* > 0.05), indicating comparable improvement rates across body sites despite early regional response differences. From week 28 onward, lesion clearance plateaued across regions.

### 3.3 Multivariate and Cox regression analysis

Univariate analysis identified four variables with *P* < 0.05 (**Supplementary Table 2**). Multivariate logistic regression analysis showed (**Table 2**) that in Model 1, patients with normal body mass index (BMI) (OR = 6.10, 95% CI: 1.54–23.96), no history of phototherapy (OR = 0.17, 95% CI: 0.04–0.71), and no previous biologic use (OR = 0.14, 95% CI: 0.03–0.62) were more likely to achieve PASI 100 at 52 weeks. These associations remained significant in Model 2 after adjustment for age and gender. Triglycerides (TG) showed no significant correlation in either model. Patients were further divided into two groups according to baseline metabolic status, and multivariate regression analysis was repeated within these subgroups (**Supplementary Table 3**). For longitudinal analysis (**Supplementary Table 4**), normal BMI (HR = 2.32, 95% CI: 1.03–5.20) was significantly associated with achievement of PASI 100 in Model 1, whereas previous biologic use, history of phototherapy, and TG showed no significant associations. In Model 2, only history of phototherapy (HR = 0.39, 95% CI: 0.15–0.98) showed a significant association with achievement of PASI 100; other variables were not statistically significant.

**Table 2:** Multivariate logistic regression analyses of patient characteristics associated with patients who achieved PASI100 at 52 weeks in comparison to those who did not.

Variables	Model 1		Model 2	
	OR (95%CI)	*P* -value	OR (95%CI)	*P* -value
Phototherapy (Y/N)	0.17(0.04-0.71)	**<0.05**	0.13(0.03-0.60)	**<0.05**
Bio-experienced (Y/N)	0.14(0.03-0.62)	**<0.01**	0.09(0.02-0.48)	**<0.01**
Baseline BMI<25 (Y/N)	6.10(1.54-23.96)	**<0.05**	6.19(1.44-26.65)	**<0.05**
TG (per SD increase in TG) #	0.69(0.35-1.35)	0.28	0.62(0.28-1.36)	0.23

BMI, body mass index; #SD: TG (SD 1.63); Bold values indicate statistical significance (*P* < 0.05).

### 3.4 Safety and drug survival

**Figure 3:**
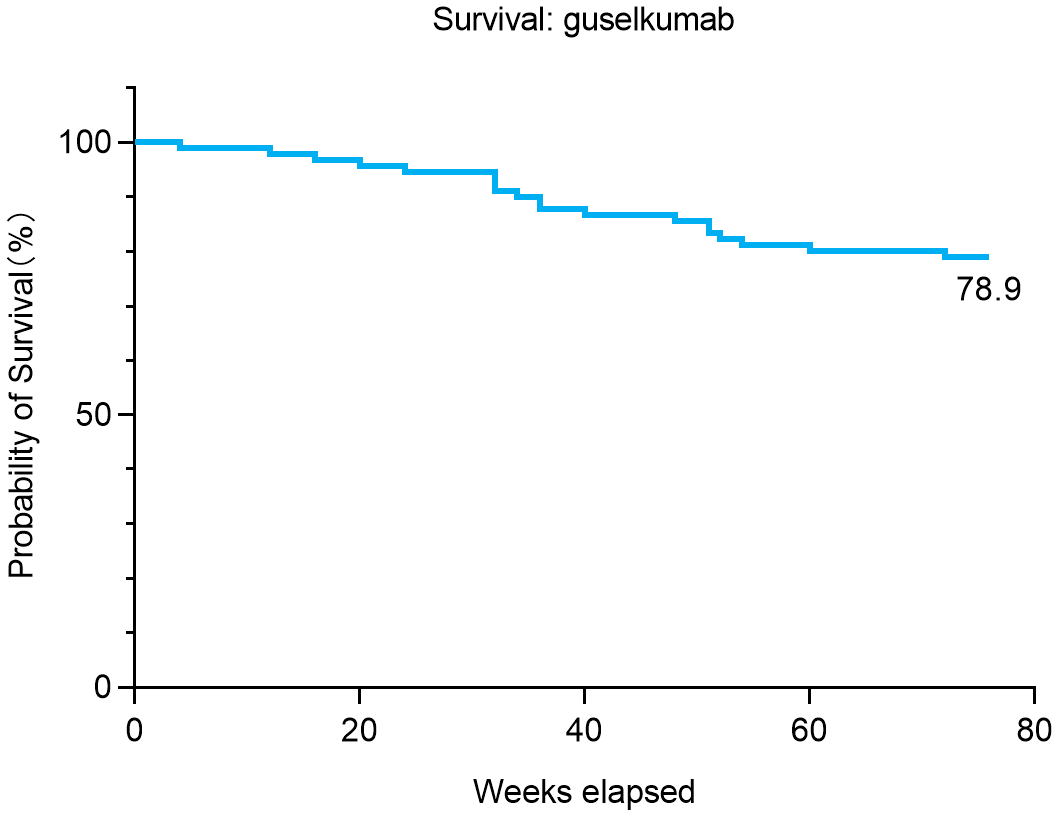
Kaplan–Meier plot of drug survival for guselkumab over 76 weeks.

In terms of safety, 32.2% (29/90) of patients experienced injection site reactions, which were the most common adverse events, followed by infections (11.1%, 10/90), cutaneous adverse events (CAEs; 5.6%, 5/90), depression (1.1%, 1/90), rhinitis (1.1%, 1/90), and other systemic diseases (1.1%, 1/90). CAEs were predominantly urticaria (3/90), followed by eczema (1/90) and acne (1/90). Only one patient discontinued treatment due to elevated liver enzymes combined with cardiac premature beats, and no other discontinuations due to adverse events were observed. Drug survival analysis showed that 21.1% (n = 19) of patients terminated follow-up early, with reasons including adverse events (n = 1, 5.3%), unsatisfactory efficacy (n = 8, 42.1%), and personal reasons (n = 10, 52.6%). The Kaplan–Meier survival curve is shown in **Figure 3**.

### 3.5 Metabolism

During the 76-week follow-up period, one patient (1.1%) initiated diabetic medication use. Paired metabolic indicator data at baseline and at 12, 28, 52, and 76 weeks after treatment in the entire cohort, the WNL group, and the AWT group are shown in **Supplementary Table 5**.

Changes in each metabolic indicator at each follow-up time point are presented in **Table 3**. In the entire cohort, mean fasting blood glucose (FBG) increased from 5.51 (0.74) to 5.65 (0.75) mmol/L from baseline to week 52 (*P* = 0.02). Subgroup analysis showed that in the WNL group, total cholesterol (TC) increased from 4.30 ± 0.61 mmol/L to 4.50 ± 0.73 mmol/L at week 28 (*P* = 0.049), and triglycerides (TG) increased from 1.07 ± 0.41 mmol/L to 1.41 ± 0.64 mmol/L (*P* = 0.009). FBG increased from 5.25 ± 0.44 mmol/L to 5.43 ± 0.45 mmol/L at week 52 (*P* = 0.01). Although these increases were statistically significant, they remained within the normal range. In the AWT group, uric acid (UA) decreased from 471.00 ± 51.06 μmol/L to 418.72 ± 64.35 μmol/L at week 52 (*P* = 0.004). At baseline, 32.7% (n = 18) of patients had UA levels exceeding the normal range, which decreased to 23.6% (n = 13) after treatment (*P* > 0.05). No significant differences were observed in other metabolic indicators at any time point or between groups. Additionally, the atherogenic index of plasma (AIP), atherogenic index (AI), lipoprotein combine index (LCI), and triglyceride–glucose (TyG) index showed no statistically significant changes before or after treatment at any time point (**Table 3**).

**Table 3:** Metabolic Parameters at Various Time Points in Patients Treated with Guselkumab, Overall and stratified by Baseline Status (WNL vs. AWT).

Parameter	Group	Baseline (Mean ± SD)	12-Week (Mean ± SD)	*P* -value	Baseline (Mean ± SD)	28-Week (Mean ± SD)	*P* -value	Baseline (Mean ± SD)	52-Week (Mean ± SD)	*P* -value	Baseline (Mean ± SD)	76-Week (Mean ± SD)	*P* -value
**TC**	Overall	4.51±1.10	4.51±1.16	0.97	4.74±0.92	4.69±0.85	0.76	4.58±0.90	4.60±0.88	0.85	4.31±0.70	4.55±0.58	0.48
	WNL	4.19±0.57	4.22±0.73	0.71	4.30±0.61	4.50±0.73	**0.049**	4.20±0.57	4.32±0.74	0.29	4.11±0.44	4.48±0.60	0.30
	AWT	6.37±1.57	6.21±1.70	0.30	5.84±0.55	5.17±1.01	0.18	5.89±0.49	5.56±0.69	0.16	None	None	None
**TG**	Overall	1.52±1.10	1.50±0.98	0.89	1.94±2.70	1.94±1.57	1.00	1.72±1.77	1.63±1.07	0.53	1.68±0.89	1.87±1.28	0.40
	WNL	1.01±0.29	1.07±0.46	0.33	1.07±0.41	1.41±0.64	**0.009**	1.02±0.33	1.15±0.70	0.16	1.09±0.25	1.13±0.63	0.72
	AWT	2.73±1.38	2.51±1.14	0.66	4.12±4.55	3.25±2.43	0.49	2.82±1.15	2.37±1.15	0.21	2.67±0.55	3.02±1.32	0.54
**HDL-C**	Overall	1.23±0.3	1.19±0.30	0.06	1.24±0.32	1.33±0.41	0.36	1.25±0.35	1.26±0.31	0.83	1.11±0.42	1.19±0.44	0.27
	WNL	1.58±0.23	1.54±0.25	0.37	1.55±0.24	1.49±0.22	0.21	1.64±0.23	1.53±0.24	0.07	1.53±0.14	1.60±0.27	0.55
	AWT	1.04±0.19	1.04±0.21	0.94	1.05±0.19	1.29±0.50	0.10	1.04±0.20	1.10±0.24	0.06	0.86±0.30	0.95±0.33	0.42
**LDL-C**	Overall	2.87±0.98	2.84±1.07	0.66	2.92±0.71	2.77±0.85	0.31	2.87±0.78	2.87±0.78	0.83	2.86±0.51	2.77±0.53	0.74
	WNL	2.59±0.53	2.54±0.69	0.74	2.59±0.53	2.55±0.74	0.80	2.51±0.47	2.61±0.64	0.22	2.62±0.31	2.71±0.60	0.77
	AWT	4.19±1.20	3.97±1.45	0.15	3.75±0.27	3.34±0.93	0.20	3.99±0.40	3.69±0.57	0.09	3.56±0.04	2.93±0.30	0.18
**FBG**	Overall	5.47±1.06	5.48±1.11	0.91	5.63±1.00	6.01±0.95	0.19	5.51±0.74	5.65±0.75	**0.02**	5.60±0.24	5.69±0.34	0.74
	WNL	5.27±0.42	5.32±0.62	0.55	5.23±0.37	5.47±0.79	0.21	5.25±0.44	5.43±0.45	**0.01**	5.33±0.68	5.65±1.17	0.45
	AWT	8.07±3.15	7.87±3.25	0.32	7.00±1.18	7.95±1.57	0.49	6.87±0.34	6.73±0.45	0.50	None	None	None
**UA**	Overall	367.31±104.45	382.04±99.96	0.17	368.73±81.48	371.27±79.36	0.82	360.22±103.14	345.85±90.77	0.14	371.33±73.18	357.44±85.26	0.41
	WNL	309.00±77.44	330.48±82.25	0.13	343.86±52.46	353.79±71.76	0.32	299.56±74.97	301.84±79.06	0.85	312.50±28.66	292.00±55.91	0.61
	AWT	480.63±51.31	464.69±76.68	0.37	477.20±40.55	425.00±80.20	0.09	471.00±51.06	418.72±64.35	**0.004**	461.67±17.04	458.33±10.97	0.82
**AI**	Overall	2.96±1.67	3.06±1.53	0.31	3.09±1.72	2.82±1.35	0.21	3.05±1.79	2.79±2.05	0.56	3.88±2.90	3.40±1.86	0.44
**AIP**	Overall	0.03±0.32	0.04±0.33	0.88	0.05±0.40	0.09±0.30	0.41	0.07±0.35	0.05±0.34	0.53	0.17±0.38	0.13±0.47	0.64
**LCI**	Overall	24.09±35.16	21.61±24.63	0.54	34.08±72.87	24.98±36.83	0.29	27.35±48.73	24.13±31.70	0.37	29.64±31.19	25.80±21.33	0.632
**TyG**	Overall	1.25±0.61	1.21±0.69	0.56	1.33±0.79	1.49±0.71	0.53	1.32±0.63	1.31±0.62	0.98	1.43±0.62	1.40±0.85	0.84

Values are presented as mean ± standard deviation (SD). TC, total cholesterol; TG, triglycerides; HDL-C, high-density lipoprotein cholesterol; LDL-C, low-density lipoprotein cholesterol; FBG, fasting blood glucose; UA, uric acid; WNL: Within Normal Limits; AWT: Abnormal without Treatment; Normal range: TC <5.2 mmol/L, TG <1.7 mmol/L, HDL-C ≥1.42 mmol/L (male)/≥1.55mmol/L (female), LDL-C <3.37 mmol/L, FBG <6.1 mmol/L, UA <428 μmol/L (male)/<357 μmol/L (female); AIP, the atherogenic index of plasma , AIP=log(TG/HDL-C);AI, atherogenic index, was defined as the ratio of non-HDL-C to HDL-C, non-HDL-C=TC-HDL-C; LCI, lipoprotein combine index ,LCI=TC∗TG∗LDL/HDL-C; TyG index, Triglyceride-Glucose Index, TyG = ln [ TG (mg/dl) × glucose (mg/dl)/2]. Units: TC, TG, HDL-C, LDL-C, and FBG in mmol/L; UA in μmol/L; Bold values indicate statistical significance (*P* < 0.05).

## 4 Discussion

Compared with other biologics, real-world studies on guselkumab in psoriasis are accumulating, although evidence in Chinese populations remains limited. This study systematically reports real-world data from 90 Chinese patients with plaque psoriasis treated with guselkumab for up to 76 weeks, confirming response differences in skin lesions across different body areas and identifying key clinical predictors of PASI 100. It also supplements data on dynamic changes in metabolic indicators, as well as CAD and insulin resistance risk indices, in Chinese patients during treatment.

In phase III clinical trials, guselkumab demonstrated consistently superior efficacy in patients with moderate-to-severe plaque psoriasis (25–28). In VOYAGE 1, guselkumab yielded significantly higher PASI 90 rates at week 16 compared with placebo (73.3% vs. 2.9%) and adalimumab (73.3% vs. 49.7(26)%). ECLIPSE confirmed guselkumab’s advantage over secukinumab in achieving PASI 90 at week 48 (29). In real-world settings, guselkumab has also shown consistent and robust efficacy (30, 31). In our study, PASI 90 and PASI 100 response rates reached 80% and 55% at week 52, confirming the long-term efficacy of guselkumab in Chinese real-world clinical practice. Additionally, severity-stratified analysis based on baseline PASI showed no statistically significant differences in efficacy between moderate and severe groups, indicating stable therapeutic effects. Moreover, the high drug survival rate of 78.9% at 76 weeks and the low incidence of adverse events confirm the good tolerability of guselkumab in real-world settings. However, most discontinuation cases were due to subjective factors (10/19), highlighting the importance of improving treatment adherence.

Previous studies have reported that psoriasis lesions in different body areas exhibit variable responses to biologic therapies, with the lower extremities often being the most refractory region, while the head and trunk typically show faster improvement (26, 32, 33). Our study confirmed the consistency of this finding in Chinese patients. This regional heterogeneity is influenced by anatomical and physiological factors, such as skin thickness and blood flow, which can affect drug penetration and immune cell activity (34–37). Furthermore, biologic switching is increasingly common in psoriasis treatment, primarily due to insufficient efficacy (primary or secondary failure), followed by drug intolerance or adverse events (38). In our study, we focused on patients who switched biologics due to inadequate efficacy. Nineteen patients with prior biologic therapy failure achieved clinical benefit after switching to guselkumab. However, the relatively small sample size necessitates further validation in larger-scale studies.

Factors affecting treatment efficacy were further explored. Multiple patient-specific factors significantly modulate treatment response in psoriasis, with obesity and biologic-experienced status often associated with reduced efficacy, while combination therapy may enhance outcomes (34, 39–45). A large real-world study by Damiani et al. showed that higher BMI was a negative predictor of secukinumab efficacy, whereas biologic-naïve individuals had superior outcomes (45). Consistent with these findings, our study identified normal BMI as a positive predictor of efficacy and prior biologic use as a negative predictor. Notably, we also observed a significant association between prior phototherapy history and reduced PASI response rates. This association likely reflects underlying disease severity rather than a direct effect of phototherapy, as patients with more severe disease are more likely to have received phototherapy. In our study, due to uncontrollable factors such as prior treatment at other institutions, we were unable to statistically evaluate the frequency and types of previous phototherapy, which may have introduced bias and warrants further investigation in prospective studies.

Regarding metabolic effects, current evidence suggests that biologic therapies for psoriasis have inconsistent effects on lipid profiles, fasting blood glucose (FBG), and uric acid (UA), with limited data available for guselkumab, particularly in Chinese populations (23, 46–51). One study reported that guselkumab significantly reduced total cholesterol and non-high-density lipoprotein cholesterol at week 12 but had no significant effects on UA or FBG (50). Another retrospective study of 49 psoriasis patients treated with guselkumab observed significant reductions in the plasma atherogenic index and TyG index at 6 months (51). Our study supplements Chinese real-world data on the metabolic effects of guselkumab and provides dynamic longitudinal data. Interestingly, TC, TG, and FBG showed slight increases at different time points in the overall cohort or WNL subgroup. However, despite statistical significance, these parameters remained within normal ranges. Moreover, the TyG index and composite lipid indices showed no statistically significant changes before or after treatment, indicating that minor changes in individual metabolic markers did not translate into increased insulin resistance or CAD risk in this cohort. In the AWT group, UA levels significantly decreased by 52.28 μmol/L at week 52, which may be attributed to normalization of keratinocyte turnover following effective psoriasis (46) control. These findings underscore the importance of individualized metabolic monitoring and suggest that long-term studies are needed to clarify cardiovascular implications.

While the core efficacy profile of guselkumab has been well established in global phase III trials and real-world cohorts, our study makes three specific contributions to the existing literature. First, we confirm previously observed response patterns—including superior efficacy in patients with normal BMI and biologic-naïve status, as well as faster scalp clearance compared with lower extremities—in a Chinese population, supporting the generalizability of these findings. Second, we provide extended follow-up data (76 weeks) on metabolic parameter changes in guselkumab-treated Chinese patients, incorporating composite cardiovascular risk indices (AIP, AI, LCI, and TyG) that have not been comprehensively evaluated in this population. Third, our regression analyses identify prior phototherapy exposure as a significant predictor of reduced likelihood of achieving PASI 100, suggesting that treatment history may be as important as baseline disease characteristics in predicting optimal outcomes. Together, these findings help bridge gaps between global evidence and Chinese clinical practice and inform personalized treatment strategies.

This study has limitations, including potential bias due to its single-center, small-sample, retrospective design, as well as unmeasured confounders such as diet, physical activity, and socioeconomic status. Future multicenter prospective studies integrating multi-omics approaches are needed to further elucidate mechanisms underlying regional response differences, the impact of treatment history, and metabolic regulatory pathways.

## 5 Conclusion

In Chinese patients with moderate-to-severe plaque psoriasis, guselkumab provides consistent and durable skin clearance over 76 weeks, with regional response differences characterized by faster improvement on the head and delayed response in the lower extremities. Superior outcomes are associated with normal BMI and biologic-naïve status. Metabolic profiling indicates no meaningful increase in cardiovascular or insulin resistance risk, and uric acid levels improve in patients with baseline elevation, supporting a favorable long-term systemic safety profile.

## Data Availability

The datasets presented in this article are not readily available because of privacy restrictions. Requests to access the datasets should be directed to feelingkun@126.com.
