# Supplementary material for: A 76-week real-world multidimensional analysis of guselkumab in moderate to severe plaque psoriasis: a retrospective cohort study based on Chinese clinical practice standards
Source: Front Immunol. 2026 Jan 27;17:1684996. doi: 10.3389/fimmu.2026.1684996 (PMC12888866; doi:10.3389/fimmu.2026.1684996)
Supplement: Supplementary file 1 [file DataSheet1.docx]

**Supplementary Table 1** Mixed-effects model comparing the rate of improvement across body regions.

| Effect | Level (Reference: Head) | Estimate | SE | *t-*value | *P-*value |
| --- | --- | --- | --- | --- | --- |
| Time |  | 0.0021 | 0.00063 | 3.35 | 0.001 |
| Region | Upper extremities | -0.0178 | 0.03337 | -0.53 | 0.593 |
|  | Lower extremities | -0.0832 | 0.03319 | -2.51 | **0.012** |
|  | Trunk | -0.0452 | 0.03357 | -1.35 | 0.179 |
| Time × Region | Upper extremities | 0.00111 | 0.00085 | 1.3 | 0.193 |
|  | Lower extremities | 0.00155 | 0.00085 | 1.82 | 0.069 |
|  | Trunk | 0.00062 | 0.00086 | 0.72 | 0.469 |

Bold values indicate statistical significance (*P*<0.05).

**Supplementary Table 2** Univariable logistic regression analyses of patient characteristics associated with patients who achieved PASI100 at 52 weeks in comparison to those who did not.

| Independent Variable | OR (95% CI) | *P* -value |  |
| --- | --- | --- | --- |
| Age | 0.99 (0.96-1.02) | 0.55 |  |
| <35 | 0.71 (0.23-2.25) | 0.56 |  |
| 35-65 | 1.77 (0.64-4.68) | 0.28 |  |
| ≥65 | 0.53 (0.12-2.31) | 0.40 |  |
| Sex (M/F) | 0.87 (0.34-2.2) | 0.77 |  |
| Age of onset (per SD increase in years) ^#^ | 0.98 (0.95-1.01) | 0.18 |  |
| Disease duration | 1.02 (0.98-1.06) | 0.37 |  |
| <5 | 0.52 (0.14-1.89) | 0.32 |  |
| 5-15 | 1.57 (0.63-3.96) | 0.34 |  |
| ≥15 | 0.90 (0.36-2.27) | 0.83 |  |
| Baseline BMI (Kg/m^2^) | 0.89 (0.80-0.99) | **<0.05** |  |
| <25 | 0.35 (0.13-0.93) | **<0.05** |  |
| 25-30 | 2.09 (0.74-5.92) | 0.17 |  |
| ≥30 | 5.03 (0.56-47.34) | 0.16 |  |
| Baseline PASI | 0.94 (0.88-1.01) | 0.08 |  |
| ≥3 and <10 | 0.46 (0.18-1.16) | 0.10 |  |
| ≥10 | 2.20 (0.86-5.61) | 0.10 |  |
| Cigarette use (Y/N) | 0.85 (0.32-2.23) | 0.74 |  |
| Family history of psoriasis (Y/N) | 1.15 (0.69-19.05) | 0.92 |  |
| Psoriatic arthritis (Y/N) | 1.15 (0.69-19.05) | 0.92 |  |
| Comorbidities | 1.19 (0.41-3.44) | 0.75 |  |
| Prior treatments  Conventional systemic agents(Y/N)  Phototherapy(Y/N)  Biologic therapy(Y/N) | 0.72(0.26-1.97)  3.54(1.23-10.16)  3.00(1.08-8.35) | 0.52  **<0.05**  **<0.05** |  |
| Glucose and lipid metabolism |  |  |  |
| FBG (per SD increase in Fasting glucose) ^#^ | 0.81 (0.49-1.36) | 0.43 |  |
| TC (per SD increase in TC) ^#^ | 0.83 (0.51-1.34) | 0.45 |  |
| TG (per SD increase in TG) ^#^ | 0.49 (0.25-0.97) | **<0.05** |  |
| HDL-C (per SD increase in HDL-C) ^#^ | 5.25 (0.95-29.18) | 0.06 |  |
| LDL-C (per SD increase in LDL-C) ^#^ | 0.89 (0.51-1.57) | 0.70 |  |

BMI, body mass index; PASI, psoriasis area and severity index; FBG, fasting blood glucose; TC, total cholesterol; TG, triglycerides; HDL-C, high-density lipoprotein cholesterol; LDL-C, low-density lipoprotein cholesterol; M/F, Male versus Female; Y/N, yes versus no; SD, standard deviation; OR, odds ratio; CI, confidence interval. ^#^SD: age of onset (SD 14.92); FBG (SD 1.00); TC (SD 1.05); TG (SD 1.63); HDL-C (SD 0.32). Bold values indicate statistical significance (*P*<0.05).

| **Supplementary Table 3** Multiple regression analysis in subgroups based on whether patients' baseline metabolism is normal. | | | | |
| --- | --- | --- | --- | --- |
| Variables | Group 1 | | Group 2 | |
|  | OR (95%CI) | *P* -value | OR (95%CI) | *P* -value |
| Phototherapy (Y/N) | 0.03(0.01-0.77) | **<0.05** | 0.25(0.06-1.10) | 0.07 |
| Bio-experienced (Y/N) | 0.12(0.01-1.34) | 0.09 | 0.19(0.04-0.98) | **<0.05** |
| Baseline BMI<25 (Y/N) | 7.27(0.48-108.20) | 0.15 | 5.24(1.17-23.56) | **<0.05** |

BMI, body mass index; Group 1: all metabolic indicators are normal; Group 2: one or more abnormal metabolic indicators; Bold values indicate statistical significance (*P*<0.05).

| **Supplementary Table 4** Cox regression analyses of patient characteristics associated with patients who achieved PASI100 at 52 weeks in comparison to those who did not. | | | | |
| --- | --- | --- | --- | --- |
| Variables | Model 1 | | Model 2 | |
|  | HR (95%CI) | *P* -value | HR (95%CI) | *P* -value |
| Phototherapy (Y/N) | 0.44(0.18-1.08) | 0.07 | 0.39(0.15-0.98) | **<0.05** |
| Bio-experienced (Y/N) | 0.51(0.20-1.33) | 0.17 | 0.42(0.16-1.10) | 0.07 |
| Baseline BMI<25 (Y/N) | 2.32(1.03-5.20) | **<0.05** | 2.14(0.92-4.99) | 0.08 |
| TG (per SD increase in TG) # | 0.83(0.53-1.30) | 0.42 | 0.71(0.41-1.24) | 0.23 |

BMI, body mass index; TG (SD 1.63); Bold values indicate statistical significance (*P*<0.05).

**Supplementary Table 5** Number of people with paired data for six metabolic parameters at baseline and at various follow-up time points in the total cohorts and subgroups.

| Group | Parameter | BL - 12W | BL - 28W | BL - 52W | BL - 76W |
| --- | --- | --- | --- | --- | --- |
| Overall | TC | 47 | 21 | 54 | 8 |
|  | TG | 47 | 21 | 54 | 8 |
|  | HDL-C | 47 | 21 | 54 | 8 |
|  | LDL-C | 47 | 21 | 54 | 8 |
|  | FBG | 44 | 18 | 53 | 7 |
|  | UA | 48 | 22 | 55 | 9 |
|  | AI | 47 | 21 | 54 | 8 |
|  | AIP | 47 | 21 | 54 | 8 |
|  | LCI | 47 | 21 | 54 | 8 |
|  | TyG | 44 | 18 | 52 | 7 |
| WNL | TC | 40 | 15 | 42 | 7 |
|  | TG | 33 | 15 | 33 | 5 |
|  | HDL-C | 39 | 18 | 42 | 6 |
|  | LDL-C | 37 | 15 | 41 | 6 |
|  | FBG | 41 | 15 | 46 | 6 |
|  | UA | 32 | 17 | 37 | 6 |
| AWT | TC | 7 | 6 | 12 | 1 |
|  | TG | 14 | 6 | 21 | 3 |
|  | HDL-C | 8 | 3 | 12 | 2 |
|  | LDL-C | 10 | 6 | 13 | 2 |
|  | FBG | 3 | 4 | 7 | 1 |
|  | UA | 16 | 5 | 18 | 3 |

WNL: Within Normal Limits; AWT: Abnormal without Treatment; Normal range: TC <5.2 mmol/L, TG <1.7 mmol/L, HDL-C ≥1.42 mmol/L (male)/≥1.55 mmol/L (female), LDL-C <3.37 mmol/L, FBG <6.1 mmol/L, UA <428 umol/L (male)/<357 μmol/L (female).

**Supplementary Figure 1**


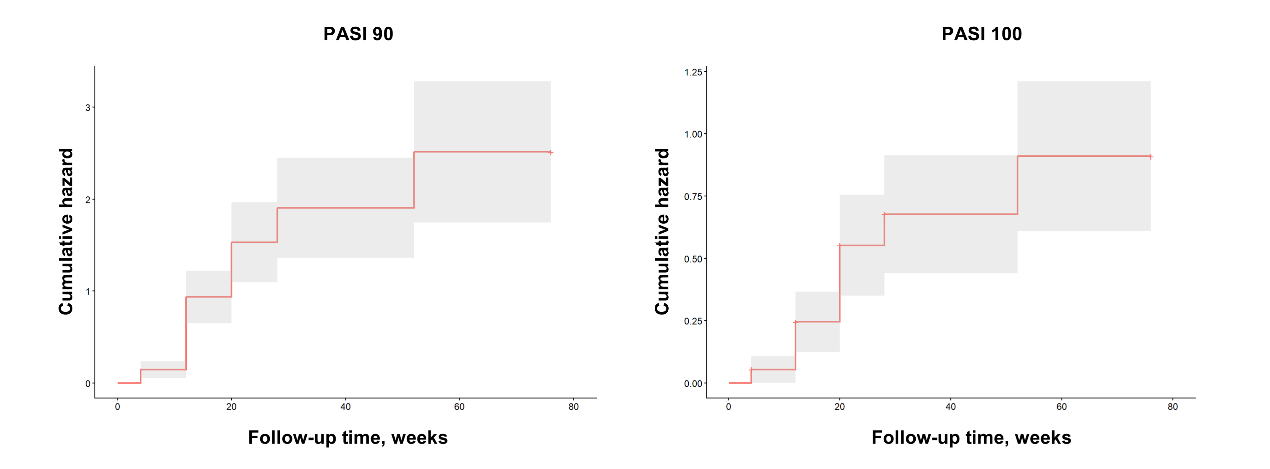


**Supplementary Figure 1** Kaplan–Meier curves for time to PASI 90 and PASI 100.

**Supplementary Figure 2**


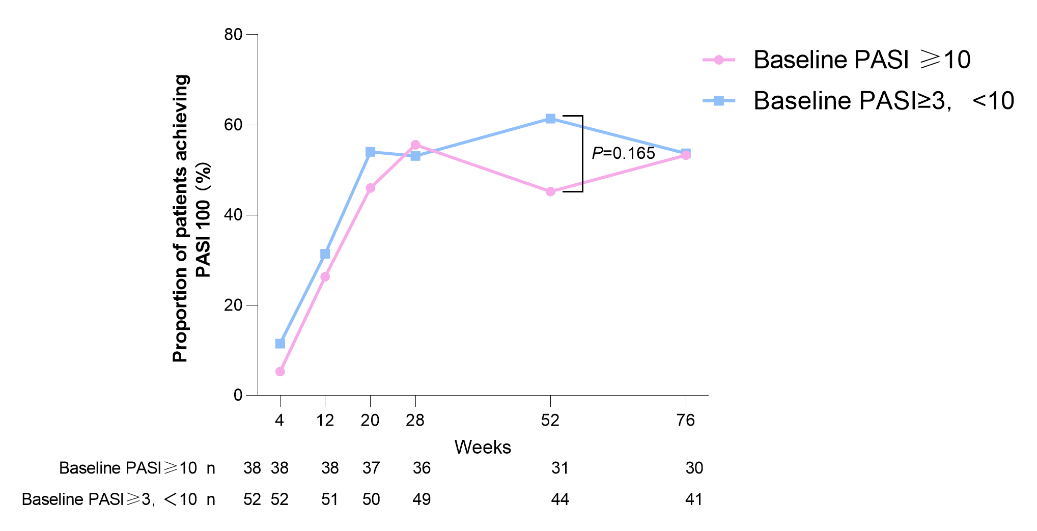


**Supplementary Figure 2** Proportion of patients with moderate or severe baseline disease severity achieving PASI 100 at week 0, 4, 12, 20, 28, 52 and 76.

**Supplementary Figure 3**


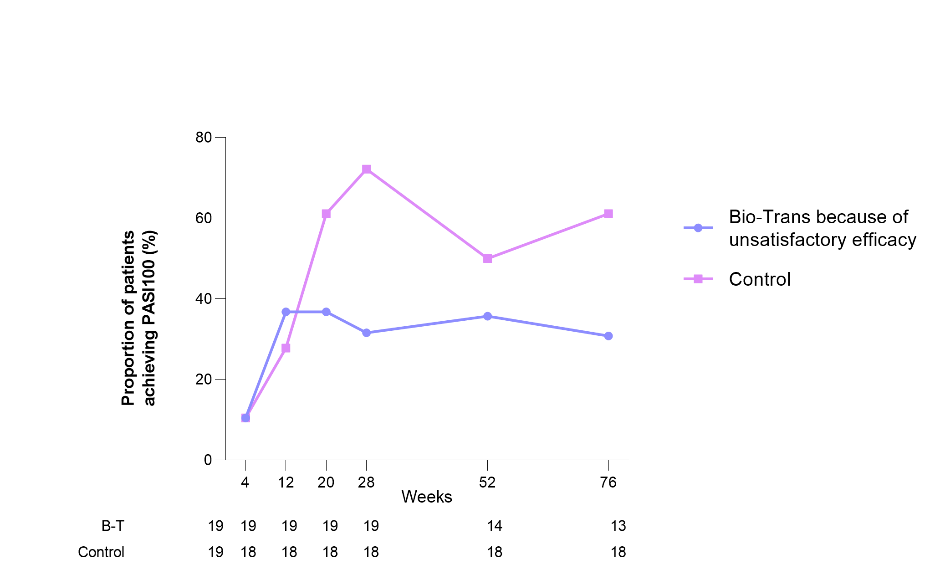


**Supplementary Figure 3** Proportion of patients achieving PASI100 at weeks 0, 4, 12, 20, 28, 52 and 76 in patients who failed prior biologics and randomly matched controls.

**Supplementary Figure 4**


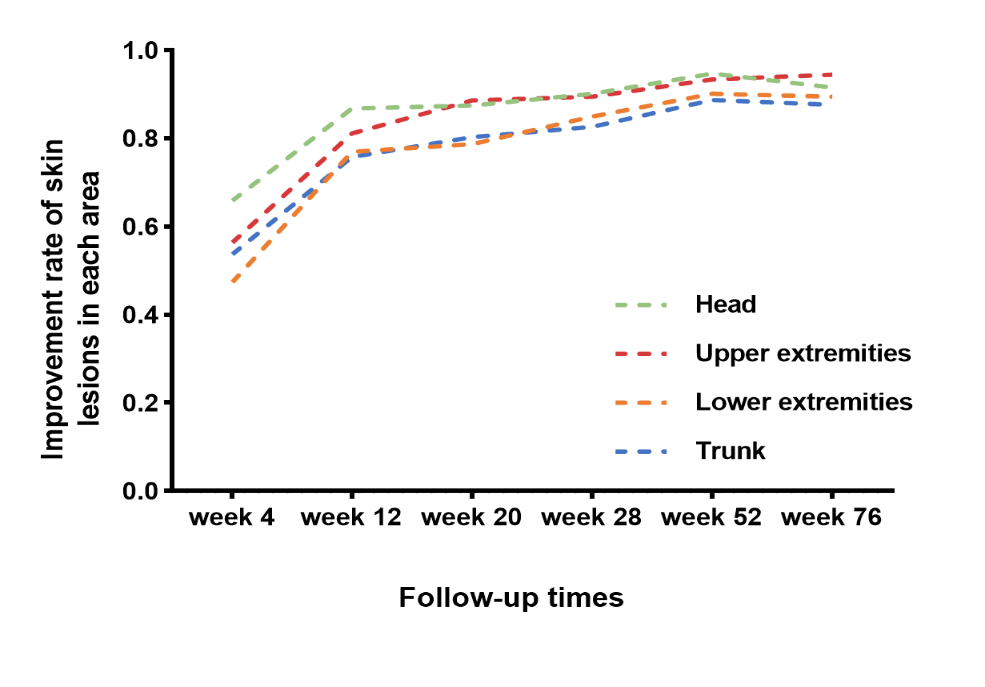


**Supplementary Figure 4** Improvement rate of skin lesions in head, lower extremities, upper extremities and trunk at weeks 0, 4, 12, 20, 28, 52 and 76.
